# The journey from genetic predisposition to medication overuse headache to its acquisition as *sequela* of chronic migraine

**DOI:** 10.1186/s10194-017-0830-2

**Published:** 2018-01-10

**Authors:** Paolo Martelletti

**Affiliations:** 1 Department of Clinical and Molecular Medicine, Sapienza University, Rome, Italy; 2 Regional Referral Headache Centre, Sant’Andrea Hospital, Via di Grottarossa, 1035, 00189 Rome, Italy

**Keywords:** Genetics, Migraine, Chronic migraine, Comorbidities, Medication overuse headache, Medication overuse, OnabotulinumtoxinA, Personalized medicine, Drug-drug-interactions

## Abstract

Migraine remains one of the biggest clinical case to be solved among the non-communicable diseases, second to low back pain for disability caused as reported by the Global Burden of Disease Study 2016. Despite this, its genetics roots are still unknown. Its evolution in chronic forms hits 2–4% of the population and causes a form so far defined Medication Overuse Headache (MOH), whose pathophysiological basis have not been explained by many dedicated studies. The Global Burden of Disease Study 2016 has not recognized MOH as independent entity, but as a sequela of Chronic Migraine. This concept, already reported in previous studies, has been confirmed by the efficacy of OnabotulinumtoxinA in Chronic Migraine independently from the presence of MOH. The consistency of the current definitions of both Medication Overuse Headache and Chronic Migraine itself might be re-read on the basis of new evidences.

## Medication overuse headache: Chasing roots and Borders

Medication Overuse Headache (MOH) is an underestimated social plague, that has always struggled to find research funds for epidemiological studies on large clinical records [1–3], probably due to non-scientific scotomata. The International Classification of Headache Disorders has interpreted MOH in an improper way through the years, and this has led to many clarifications and criticisms [4, 5]. In fact, it is considered a headache secondary to the pharmacological abuse itself, without any consideration of the fact that MOH roots on Chronic Migraine (CM) [6]. Therefore, it must be considered as a complication of CM only. Rehabilitation from drug abuse, in whichever modality it is carried out, produces de facto a return to CM [7]. The same therapies applied today to CM are in fact efficacious with or without the presence of MOH, and an artificial distinction of a classification does not distinguish their efficacy [8–10].

Furthermore, we know that when migraineurs, whose genetic base remains yet undefined, despite the scientific efforts [11], develop CM plus Medication Overuse (MO) do not show univocal genetic patterns. Studies in this sector are at least jeopardized, and results correlate on various polymorphisms not always assonant, if we consider their action as drivers on possible physio-pathological patterns [12–15]. Many refined functional neuroimaging studies applied to MOH undergo the same jeopardized criteria [16–18].

So, if we look for a generator of CM complication in CM plus MO we will have to look at the comorbidities spectrum, like depression, visceral pain, fibromyalgia, orofacial pain, temporo-mandibular disorders, myofascial trigger points that act on migraine incrementing its disability and an uncontrolled multiple need of analgesics [19]. This additional use disadvantages only migraine, since the patient with other osteoarticular pathologies who frequently uses analgesics and antimigraine drugs, if not migraneurs, will never develop any MO(H), ever. [19].

## Changing the classification of MOH as *sequela* of CM

MOH doesn’t have a genetic predisposition, then. Contradictory and varied studies did not lead to common denominator for a simple reason: MOH is not an entity independently classifiable, as the International Classification of Headache Disorders leads us to thin. It is a simple sequela, a complication of CM, as we affirmed in 2011 [4, 5] and now validated in the recent Global Burden of Diseases 2016 (GBD2016) [19]. GBD2016 is to be considered today as the driving light on confirming, through epidemiology and disability parameters, that MO(H) is only a *sequela* of CM and must be summed in this clinical context adding a stronger value to migraine itself, now ranked second place, behind the low back pain only [20].

Therefore, the treatment for CM, now based on the use of OnabotulinumtoxinA, should include the management of its *sequela*, making suddenly drop all the puzzles arisen after the registration studies, in which 2/3 of enrolled CM patients had a co-diagnosis of MOH. Such uncertainties have fallen with the following real-world studies with OnabotulinumtoxinA for CM where all treated patients were suffering from MO. The drug efficacy contextually reduced, and it couldn’t be otherwise, headache days and consequently the excessive intake of medication.

**Fig. 1:**
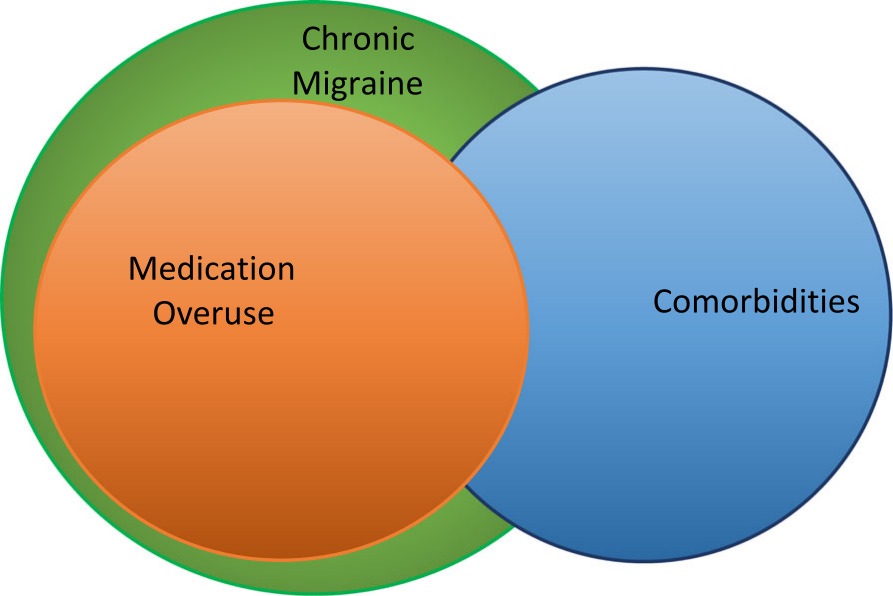
Putative overlapping of Chronic Migraine, Medication Overuse and Migraine Comorbidities

The necessity of a definitive redefinition of this clinical situation of *Chronic Migraine plus Medication Overuse* [4] overwhelmingly arises. Figure 1 pieces together these clinical evidences.

We could shortly witness a new pharmacological era based on the use, in migraine treatment, of monoclonal antibodies against Calcitonin-Gene-Related-Peptide or its receptors, that if properly used in intercepting the evolution from high-frequency migraine towards its chronic phase will be able to reduce, in a decade, CM and its complication, MO [21].

Another matter recently opened on the solidness of the wall that supports the definition of chronic migraine, the 15 days per month of migraine, seems to waver on the basis of the brilliant study winner of the 2017 Greppi Award: the natural fluctuation above and below this barrier strongly challenge the conceptual modality of defining a form of migraine as chronic [22].

## Getting to the bottom of MO through personalized medicine

**Fig. 2:**
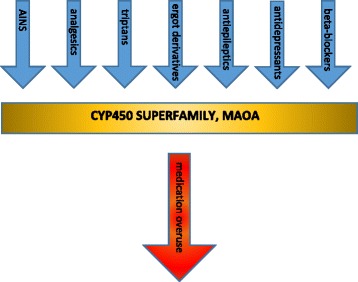
Drug-drug interactions between preventative and acute migraine medications. The reported classes of drugs compete for the same metabolic pathway, and this represents the most common causes of drug reduction of efficacy and/or toxicity (for details please refer to ref. [23], specifically to tables 1 and 2), leading to medication overuse. Legenda AINS: anti-inflammatory non-steroidal drugs; CYP450: cytochromes P450 (accounting for the metabolism of more than 70% of prescribed drugs); MAOA: monoaminoxidase-A

We think today that MO originates from a numeric calculation and this causes overuse in a *quasi*-mysterious way. This cannot be all true. In fact, migraine experts prescribe acute and preventative therapies to mitigate pain and the symptomatology of existing comorbidities [23]. Real world setting migraine patients might also have other pathologies, not comorbid with migraine, that require additional therapies. This complex treatment may lead to many drug-drug interactions (DDIs), risking to develop not only Adverse Events (AEs) but also to reduce the clinical efficacy of the administered drug. This is particularly true in the case of migraine acute drugs leading to MO [24]. Genotype and phenotype also have an influence in the development of DDIs, therefore knowing them is crucial in choosing the best acute/preventative treatment and the appropriate drug interactions. The DrugBank, SuperCyp, PharmaGkb [23] are data banks containing all the potential genotype-phenotype correlations in which it is possible to observe all the acknowledged DDIs and, knowing these, to recommend a tailored treatment avoiding metabolic clashes: in fact, more than 70% of prescribed drugs compete for the same metabolic pathway, the superfamily of cytochrome CYP450, and this represents the most common cause of drug reduction of efficacy and/or toxicity (Fig. 2). By avoiding this cross-competition we will be able to control this reduction of efficacy or drug toxicity and, therefore, the risk of MO. The Personalized Medicine model is particularly valid in migraine, where there is special need for multiple therapies due to the high number of comorbidities [25, 26]. With this clinic-molecular-biology-based approach, we will be able to obtain the better therapeutic performance, avoid DDIs, and practically combine acute, preventative and comorbidities treatments as well as additional sided therapies [26, 27]. Personalized Medicine is the future for safe migraine treatment.

## Final re-reading

Migraine is the second burdensome illness, if considered in terms of prevalence and disability. Its development in chronicity highlights important personal, organizational and health care problems. The distinction between CM and MOH has created cultural barriers in the management and rehabilitation. The natural fluctuations of headache days challenge the intrinsic definition of CM as we know it, and this clashes with the concept that migraine is a solidly progressive disorder. It is then necessary to bring MOH back to the simple definition of CM complication, where various genetic factors yet to be defined in large scales, multiple comorbidity factors and inadequate lifestyles might explain its development, and to re-read the CM core. The possibility in a near future to intercept migraine evolution from high frequency onward through the new pharmacological class of the monoclonal antibodies towards Calcitonin-Gene-Related-Peptide or its receptors might reduce its progression towards its fearsome complication, medication overuse.
